# Low-Light Image Enhancement Guided by Hybrid Explicit-Implicit Semantics

**DOI:** 10.3390/s26217019

**Published:** 2026-11-06

**Authors:** Zanhao Liang, Ruixiao Huang, Xuhui Ma, Binbing Liu

**Affiliations:** School of Optical and Electronic Information, Huazhong University of Science and Technology, Wuhan 430074, China; m202373113@hust.edu.cn (Z.L.); d202581511@hust.edu.cn (R.H.); chandler9488@gmail.com (X.M.)

**Keywords:** low-light image enhancement, semantic guidance, image restoration

## Abstract

Low-light image enhancement (LLIE) is fundamentally a restoration problem rather than a brightness adjustment task, as it aims to recover the underlying scene information from images captured under low signal-to-noise ratio conditions. However, most existing methods adopt globally uniform enhancement schemes and overlook the degradation discrepancies across semantic regions, limiting overall performance. Recent semantic-guided LLIE methods often neglect how low-light degradation impairs the fidelity of semantic priors, preventing them from exploiting semantic information. To address these issues, we propose an implicit semantic guidance branch that employs a Light-up Module and a Feature Alignment Module to obtain accurate semantic features and adaptively integrate them into the feature representation space. Furthermore, we introduce an explicit semantic guidance branch that constructs a Semantic-Guided Pixel Consistency Loss using accurate semantic maps obtained by SAM 3, mitigating segmentation errors caused by cross-dataset label space discrepancies. Additionally, we develop a carefully designed low-light degradation model to synthesize training data and adopt a pre-training strategy, enabling the network to learn fine-grained semantic-aware brightness mappings. Based on these components, we develop a hybrid explicit–implicit semantic guidance framework. Extensive experiments demonstrate that our method outperforms state-of-the-art approaches in image quality, noise suppression, and structure preservation.

## 1. Introduction

Images captured in low-light conditions typically undergo severe visual degradation in the form of low contrast and unavoidable noise, which severely impairs their aesthetic and practical value due to substantial brightness deterioration and detail degradation. Furthermore, such degraded images impair the performance of high-level visual models trained on high-quality images and may even render them inapplicable [1]. Consequently, various computer vision applications demand high-performance LLIE algorithms, since the quality of enhanced images can substantially affect the performance of downstream tasks such as object detection.

Traditional low-light image enhancement methods mainly adopt handcrafted prior approaches such as histogram equalization, Retinex-based methods and gamma correction. They exhibit limited robustness against color distortion and noise interference, as LLIE is essentially a nonlinear problem with unknown and complex degradation characteristics.

Recently, deep learning-based methods have attracted widespread attention in the field of low-light image enhancement. Researchers have developed numerous data-driven network approaches. Unlike traditional methods relying on handcrafted priors, these deep learning methods exhibit powerful nonlinear fitting ability and superior capability in modeling complex degradation patterns.

The majority of deep learning-based LLIE methods aim to learn the nonlinear mapping between a low-light image and its normal-light counterpart, including end-to-end frameworks [2] and Retinex-based frameworks [3]. With the advancement of deep learning, recent methods have progressively evolved from CNN-based to Transformer-based architectures, allowing them to capture more complex luminance mappings and thereby further improve their performance. However, as these methods overlook the substantial variation in degradation across distinct semantic regions, they tend to enforce globally uniform brightness enhancement, which inherently restricts model performance.

Accordingly, recent image restoration methods have increasingly utilized semantic information to guide network training, typically in the form of semantic features or semantic maps derived from a pre-trained semantic segmentation model. For instance, Fan et al. [4] leveraged semantic maps and integrated them into the image feature space to improve image quality, while Zheng [5] employed a novel semantic loss to enforce semantic consistency in the enhanced images. Furthermore, to capitalize more effectively on semantic information, Wu et al. [6] proposed a semantic-aware knowledge-guided framework that not only introduced a semantic-aware module to efficiently leverage semantic features extracted by a segmentation model to enhance existing methods, but also incorporated a semantic-guided color histogram loss and a semantic-guided adversarial loss based on semantic maps, thereby further enforcing semantic consistency in the enhanced images. Li et al. [7] extracted semantic features from images using a pre-trained CLIP model and integrated them into the feature space of the discriminator, thereby guiding the discriminator to perceive semantic information and reducing artifacts in the outputs of GAN-based image super-resolution networks. However, these methods ignore the fact that LLIE itself inherently serves as an upstream task for high-level vision. Directly applying high-level models such as segmentation networks to degraded images inevitably yields impaired features, even though data augmentation with noise and other degradations is commonly employed during their training. Hence, incorporating these degraded features offers no assurance of performance gains.

In summary, LLIE methods based on semantic guidance currently face two major limitations. First, directly applying pre-trained semantic or instance segmentation models to low-light images yields semantic maps or semantic features that are severely degraded, rendering the experimental results inconsistent. Second, even when semantic-guided losses are designed using maps inferred from clean images via semantic segmentation models, the discrepancy between the label spaces of segmentation datasets and LLIE datasets may introduce segmentation errors into the semantic maps, thereby impairing the effectiveness of the corresponding losses. 

**Figure 1:**
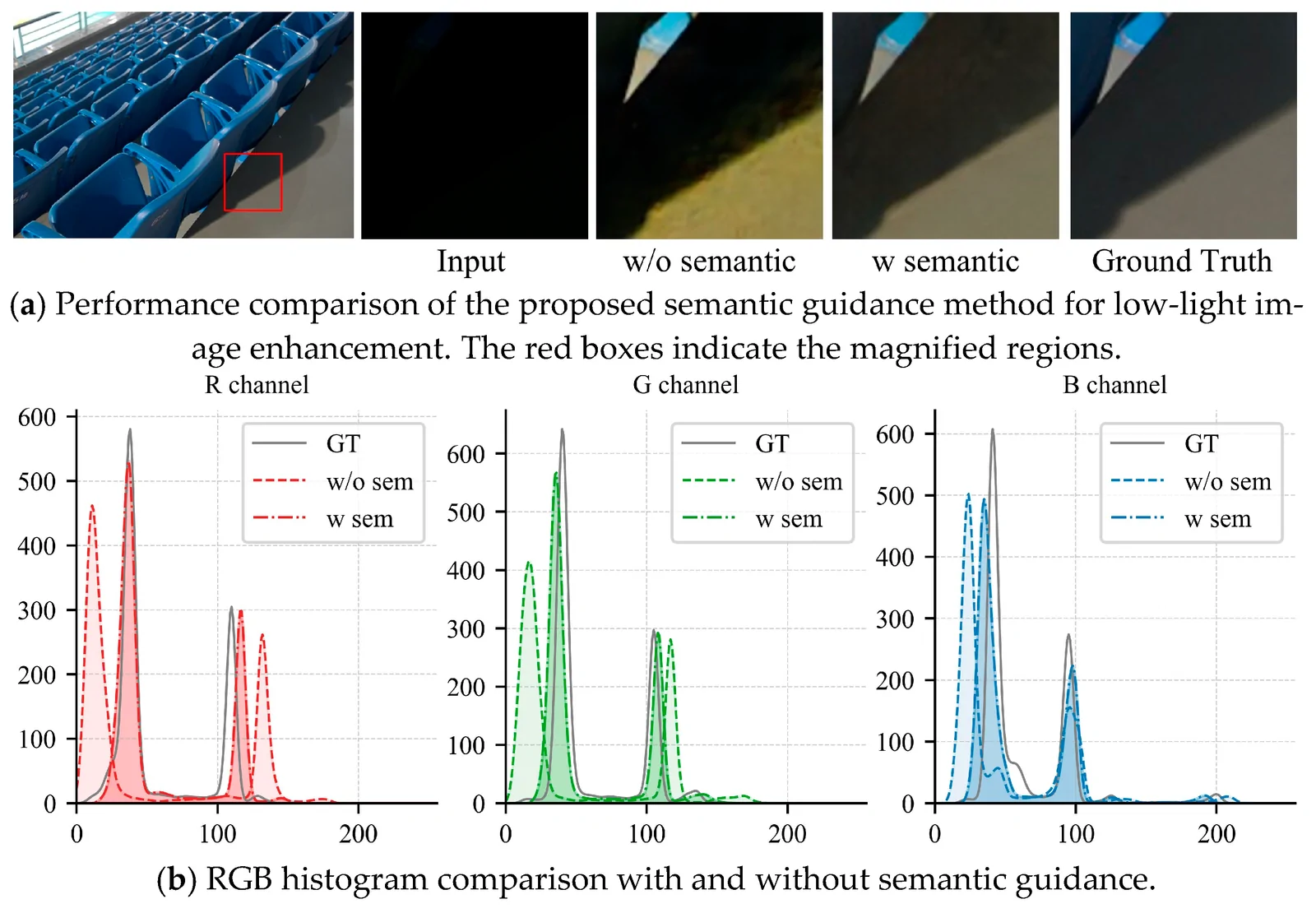
Motivation and effect of semantic guidance. (**a**) The enhancement results without semantic guidance show color distortion. (**b**) Our proposed semantic guidance method demonstrates excellent color correction performance in histogram analysis, using the images in (**a**).

To address these issues, we propose a method termed Low-light Image Enhancement Guided by Hybrid Explicit–Implicit Semantics. By integrating both implicit and explicit guidance, we establish a hybrid explicit–implicit semantic guidance framework. As shown in Figure 1, the proposed semantic guidance method performs favorably in color correction, with visualization results and histogram analysis shown in Figure 1a and Figure 1b, respectively. In this framework, implicit semantic guidance leverages a pre-trained CLIP image encoder [8] with remarkable zero-shot capability to extract semantic features from low-light images.

Compared to those obtained from pre-trained semantic segmentation models, the semantic features derived by CLIP from low-light images preserve substantially higher content fidelity [9]. Then, we design a Light-up Module to refine the accuracy of these semantic features, and a Feature Alignment Module to learn the mapping between features extracted by encoders designed for different computer vision tasks, thereby effectively integrating semantic features into the feature representation space. Explicit semantic guidance, on the other hand, refers to the use of semantic maps inferred by SAM 3 from clean images to formulate semantic losses. SAM 3, with its powerful open-vocabulary segmentation and text-prompt segmentation capabilities, offers highly flexible semantic segmentation. Furthermore, we design a low-light degradation model to synthesize simulated data. This enables us to select clean images with appropriate labels for training. We employ a pre-training stage on the synthetic data with semantic loss constraints, thereby guiding the network to learn degradation-aware representations across different semantic regions. As shown in Figure 1a, semantic guidance enables the preservation of fine textures in diverse regions, overcoming the common limitation of global uniform enhancement that leads to uneven denoising and partial over-smoothing. The main contributions of this paper are as follows:

- We propose a hybrid explicit–implicit semantic guidance framework for low-light image enhancement, focusing on improving the reliability of semantic priors and their effective utilization in image restoration.
- For implicit semantic guidance, we employ a contrastively pre-trained CLIP image encoder, which provides more degradation-robust semantic features. We further introduce a Light-up Module (LM) and a Feature Alignment Module (FAM) to improve semantic feature extraction and alignment with the restoration feature space.
- For explicit semantic guidance, we develop a semantic-aware pre-training strategy using clean images with a consistent semantic label space. A physically motivated degradation model synthesizes paired low-light data, while the proposed Semantic-Guided Pixel Consistency Loss leverages semantic maps generated by SAM 3 from clean images to provide differentiated supervision across semantic regions, encouraging region-adaptive restoration.

## 2. Related Work

### 2.1. Learning-Based Methods

Deep learning-based methods have demonstrated strong capabilities in modeling complex low-light degradation. Lore et al. [2] proposed LLNet, the first deep learning-based network for low-light image enhancement. Wei et al. [3] proposed the first Retinex-based network, Retinex-Net, which separately trains the decomposed reflectance and illumination components. However, because the network fails to adequately fit these decomposition functions, the output images suffer from severe artifacts, retain substantial noise, and exhibit noticeable color distortion. Owing to the superior capability of Transformer-based models in capturing long-range dependencies, SNRNet [10] incorporates a Transformer-based long-range branch to restore features in low-SNR regions, achieving impressive denoising performance. However, it tends to excessively smooth high-frequency details. Compared with SNRNet, which merely incorporates a Transformer-based module, Retinexformer [11] is the first Transformer-based algorithm for LLIE grounded in Retinex theory. By further refining the Retinex model, it learns illumination priors and leverages them to guide the network training, thereby raising the upper bound of LLIE performance. However, it ignores the intricate nonlinearity of illumination and estimates the prior using only a simple depthwise separable module, which can easily lead to overfitting.

Frequency-domain methods have also been explored for low-light image enhancement, typically employing Fourier or wavelet transforms to facilitate illumination restoration, noise suppression, and detail recovery [12,13]. However, noise and image details may occupy overlapping frequency bands, making it difficult to distinguish degradation from structural information based solely on frequency characteristics. Furthermore, frequency decomposition alone has limitations in capturing semantic information, which cannot be directly associated with a specific frequency band but instead depends on features and their relationships across multiple spatial scales.

Furthermore, owing to the impressive success of Denoising Diffusion Probabilistic Models in image generation, a growing body of diffusion-based generative models for LLIE has recently been proposed, which have yielded more favorable outcomes in heavily noise-degraded regions under extreme low-light conditions. To mitigate the hallucination artifacts that are difficult to control in diffusion-based models, Hou et al. [14] formulated a global structure-aware regularization that facilitates the preservation of complicated details. The method in [15] combines Retinex theory with the powerful generative capability of diffusion-based models, thereby integrating the advantages of both physical and generative approaches. However, existing methods tend to adopt global uniform optimization schemes that restrict their performance upper bound while ignoring the degradation heterogeneity across diverse semantic scenes.

### 2.2. Semantic Guidance-Based Methods

To preserve semantic information during low-light image enhancement, Zheng [5] was the first to leverage an unsupervised semantic segmentation model to formulate a semantic loss as an auxiliary objective. Li et al. [16] utilized semantic segmentation maps as priors to guide the restoration of reflectance maps decomposed by a Retinex network. Liang et al. [17] proposed a semantic brightness consistency loss to constrain the model to learn the luminance distributions across different semantic regions. Instead of using semantic loss, Fan et al. [4] incorporated semantic maps inferred by a semantic segmentation model on low-light images as semantic priors into the image feature representation space, thereby endowing the model with semantic-aware capability. Furthermore, Wu et al. [6] proposed a semantic-aware knowledge-guided framework, which involves fusing semantic features from a pre-trained semantic segmentation model via a Transformer-based module, and designing a semantic-guided color histogram loss and a semantic-guided adversarial loss using the semantic maps generated by the same model. Unlike methods that rely on semantic maps produced by pre-trained segmentation models, Li et al. [18] exploited a high-frequency physical prior that embraces rich semantic information and adaptively assists the learning process. Generated by SAM [19] under diverse scenarios, these priors benefit from SAM’s zero-shot capability, thereby mitigating inaccuracies in semantic guidance.

However, these methods overlook the fact that semantic segmentation models cannot reliably interpret low-light images. Despite these advances, existing semantic-guided LLIE methods still face two major challenges. First, semantic priors extracted directly from low-light images may be unreliable due to severe illumination degradation, as pre-trained semantic models are primarily developed using high-quality images. Second, closed-set segmentation models may produce inaccurate semantic maps due to label space mismatches with LLIE datasets, thereby weakening semantic supervision.

To address these limitations, we propose a hybrid explicit–implicit semantic guidance framework. The implicit semantic guidance branch employs CLIP, together with the proposed Light-up Module and Feature Alignment Module, to improve semantic feature reliability and alignment. Meanwhile, the explicit semantic guidance branch utilizes semantic maps generated by SAM 3 from clean images and introduces a Semantic-Guided Pixel Consistency Loss during synthetic low-light pre-training to provide differentiated supervision across semantic regions.

## 3. Methods

**Figure 2:**
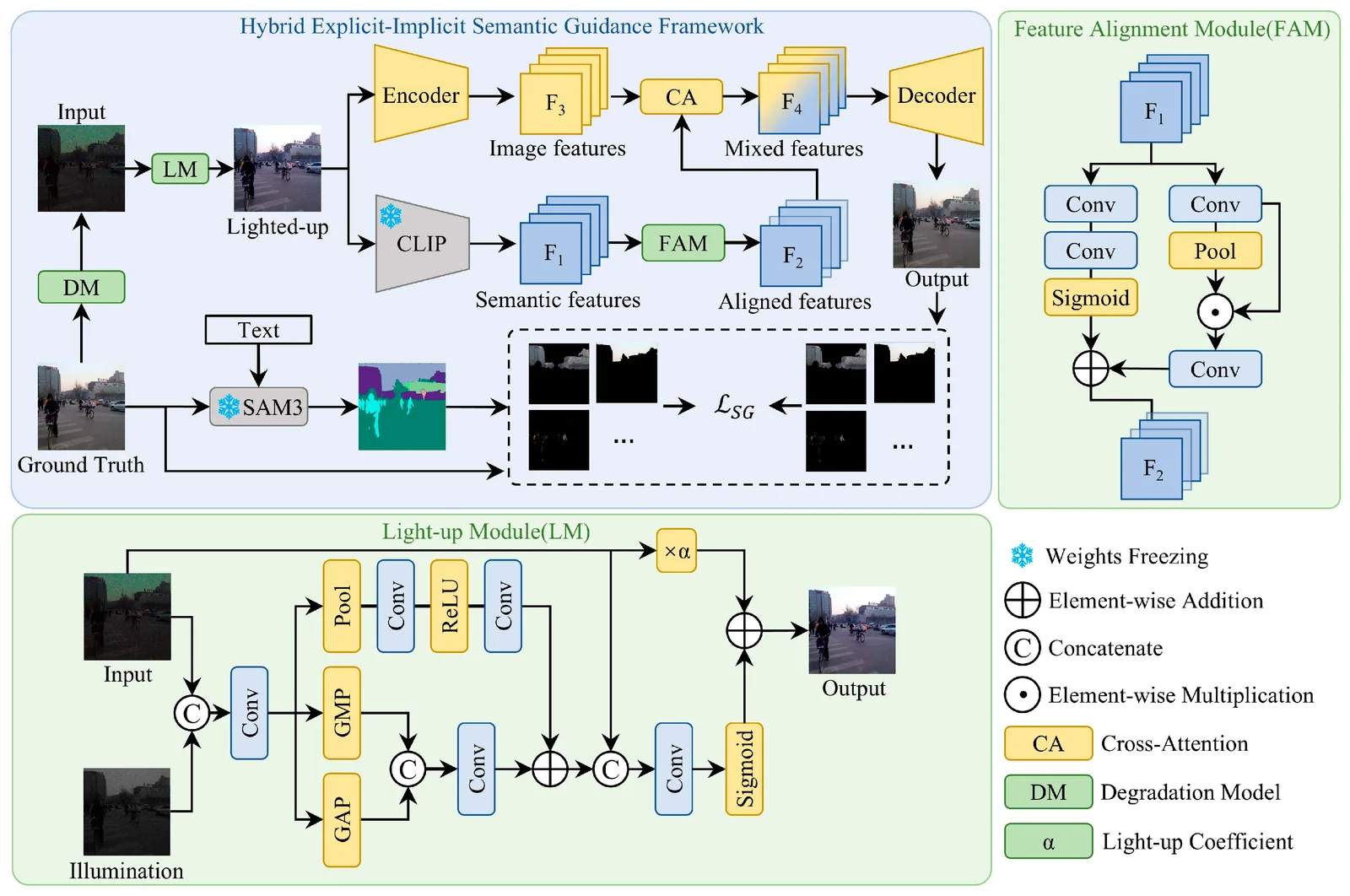
The overall structure of our proposed method. It consists mainly of an implicit branch and an explicit branch. The implicit branch utilizes the proposed Light-up Module (LM) and the Feature Alignment Module (FAM) to enhance the semantic features extracted by the CLIP image encoder. The explicit branch leverages a Semantic-Guided Pixel Consistency Loss, constructed from semantic maps inferred by SAM 3, to supervise the training process.

As illustrated in Figure 2, our framework consists of two complementary semantic guidance branches, namely implicit and explicit semantic guidance. In the implicit semantic guidance branch, the input low-light image is first processed by the Light-up Module to mitigate the impact of illumination degradation on subsequent feature extraction. The resulting image is then fed into the restoration encoder and pre-trained CLIP image encoder to extract image features and semantic features, respectively. To bridge the representation gap between features extracted by different encoders, we introduce a Feature Alignment Module to refine and align the semantic features. Subsequently, the aligned semantic features are integrated with the image features through the semantic-aware embedding module adopted from [6], and the fused features are passed to the decoder to reconstruct the enhanced image.

In the explicit semantic guidance branch, which aims to provide semantic-aware supervision, we propose the Semantic-Guided Pixel Consistency Loss based on reliable open-set semantic segmentation maps generated by the pre-trained SAM 3 model. However, existing LLIE datasets are primarily designed for image enhancement rather than semantic segmentation, and their diverse scene compositions make it difficult to establish a unified semantic label space for reliable segmentation. Therefore, we adopt the RGB500 dataset, which contains clean images with suitable semantic categories, to generate synthetic low-light training data. Following the physical imaging principles and noise modeling techniques in [20,21], we construct a low-light degradation pipeline to simulate realistic degradation processes and produce paired samples for pre-training. During fine-tuning, the network is optimized without semantic segmentation supervision. At inference, neither the degradation pipeline nor SAM 3 is required, while the implicit semantic guidance branch remains active to guide image restoration.

### 3.1. Implicit Semantic Guidance Branch

In the implicit semantic guidance branch, a pre-trained model is employed to extract semantic features that integrate semantic information into the image representation, thereby equipping the network with semantic-aware capability. However, even advanced pre-trained models struggle to extract accurate semantic features from degraded images such as low-light images. Furthermore, the image features extracted by different encoders optimized for distinct tasks exhibit significant misalignment. To address these challenges in leveraging semantic features for preserving image semantics, we introduce a Light-up Module and a Feature Alignment Module to pre-illuminate the input image and align image features from different encoders, respectively.

#### 3.1.1. Semantic Feature Encoder

**Figure 3:**
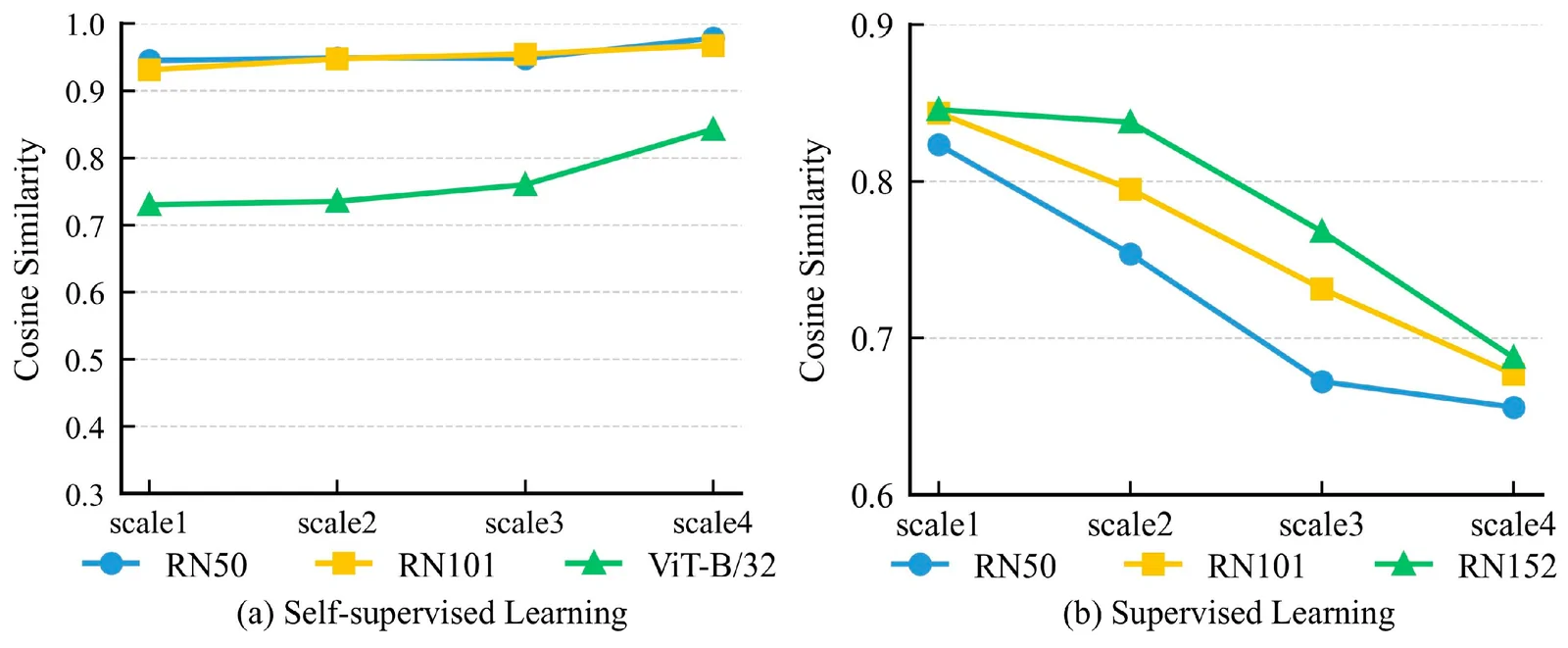
(**a**) Semantic feature similarity between low-light images and their normal-light counterparts obtained by contrastively trained CLIP image encoders (ResNet and ViT versions). (**b**) Semantic feature similarity between low-light images and their normal-light counterparts for a supervised ResNet [22].

Inspired by [9], we extract the feature maps preceding each average or attention pooling operation in the image encoder from both low-light images and their normal-light counterparts. This yields four multi-scale feature maps for each image, denoted as $F_{i}\in R^{\frac{H}{2^{i}}\times \frac{W}{2^{i}}\times 2^{i}C}$, *i* ∈ {2,⋯ 5}, where *H* × *W* are the spatial dimensions of the input image, and *C* is the base channel number. We then compute the cosine similarity between the corresponding feature maps to assess the robustness of the features extracted from low-light images. As shown in Figure 3b, the semantic features extracted by the supervised ResNet from low-light images and their normal-light counterparts exhibit a low cosine similarity, indicating that low-light images hinder semantic feature extraction by networks that rely on clean images. This is mainly because the supervised ResNet tends to rely on low-level cues such as texture and edges for classification, which are severely degraded under low-light conditions. Cheng et al. utilized CLIP [9] to suppress out-of-distribution (OOD) noise and showed that the CLIP image encoder can extract degradation-robust features, maintaining high consistency between the representations of degraded images and their clean counterparts. Motivated by this observation, we investigate whether this robustness extends to low-light images. As shown in Figure 3a, the CLIP image encoder exhibits similar characteristics, producing highly consistent feature representations for low-light images and their normal-light counterparts. Furthermore, benefiting from the contrastive learning strategy and image-text matching constraints, the CLIP image encoder can extract implicit features that contain rich semantic information. Therefore, we adopt the CLIP image encoder to extract semantic features, thereby guiding the model to perceive semantic information during image enhancement. Notably, the pre-trained CLIP image encoder is also employed during inference to extract semantic features for subsequent feature alignment and integration.

#### 3.1.2. Light-Up Module

To extract semantic features from a pre-trained model, some works [6,18] directly feed low-light images as input into the pre-trained model. However, due to low contrast and severe noise corruption, the visual information in low-light images becomes ambiguous even degraded. No matter how advanced the model’s capability in extracting semantic features, it can only obtain incomplete semantic information from low-light images to serve as the prior for guiding semantic perception, which ultimately limits performance gains. Therefore, it is essential to improve the accuracy of semantic features before they are utilized. Cai et al. [11] adopted depthwise separable convolution to estimate an illumination map for enhancing the input image. However, relying on depthwise separable convolutions for illumination estimation may limit the ability to capture complex nonlinear illumination variations. To address this limitation, we propose a Light-up Module (LM), as illustrated in Figure 2, to improve the illumination of low-light images before semantic feature extraction, thereby enabling the pre-trained encoder to obtain more reliable semantic representations. By substituting convolutional attention for Transformer self-attention, the Light-up Module learns complex nonlinear luminance mappings with lower computational cost and generates an attention map of illumination. Specifically, we replace the matrix multiplication in self-attention with concatenation and substitute the feed-forward network with convolution layers. As shown in Table 1, compared with the Transformer self-attention module, LM reduces the parameter count from 0.0060 M to 0.0034 M and the computational cost from 0.2193 GFLOPs to 0.1011 GFLOPs, corresponding to reductions of 43.33% and 53.90%, respectively. All FLOPs are calculated using an RGB input image of size 192 × 192.

The result is then added to the input image pre-amplified by the lighting coefficient to produce the final light-up map. Compared with the light map in [11], scaling the input coefficient beforehand inherently avoids negative pixel values in the light-up map caused by overfitting.

#### 3.1.3. Feature Alignment Module

The CLIP image encoder extracts semantic features from the low-light image pre-illuminated by the Light-up Module. However, due to the inherent task discrepancy, these semantic features are not well aligned with the image features produced by the LLIE encoder. To align the latent space of semantic features with that of image features, we propose a Feature Alignment Module (FAM) to refine the semantic feature maps, as illustrated in Figure 2. This module employs a convolutional branch to learn the latent space mapping between the two types of features. In addition, to enable the model to better utilize different semantic features, a channel attention branch is designed to adaptively assign importance weights to the semantic features, thereby selecting more beneficial features while suppressing the influence of those that have been severely degraded.

To further facilitate feature alignment, we introduce a cosine similarity-based feature consistency loss to supervise the aligned features produced by the Feature Alignment Module. This loss encourages the aligned features to maintain consistency with the reference features and is formulated as follows:

(1) $$L_{\mathrm{align}}=\;1-\frac{\langle F_{\mathrm{align}},F_{\mathrm{ref}}\rangle }{{\left\|F_{\mathrm{align}}\right\|}_{2}{\left\|F_{\mathrm{ref}}\right\|}_{2}},$$

where *F_align_* denotes the features produced by the Feature Alignment Module, *F_ref_* represents the reference features directly extracted by the CLIP image encoder without passing through FAM. Both feature representations are flattened into vectors before computing their cosine similarity.

Finally, we employ the semantic-aware embedding module proposed in [6], which leverages a cross-attention mechanism to integrate semantic features into the latent space of image features, thereby guiding the model to perceive semantic information.

### 3.2. Explicit Semantic Guidance Branch

Semantic maps obtained by pre-trained semantic segmentation models are always used to divide the image into patches with different labels. Each region corresponds to a specific semantic category. Hence, many studies employ semantic maps to formulate semantic losses that enforce pixel-level semantic consistency. However, these works use semantic segmentation models lacking zero-shot capacity, so they cannot obtain correct segmentation results because the label spaces of semantic segmentation datasets and LLIE datasets are different. Furthermore, low-light image enhancement datasets are not designed for semantic segmentation tasks. It is difficult to find a suitable label set for a pre-trained semantic segmentation model, especially for indoor scenes filled with various man-made objects. To address these challenges, we adopt the RGB500 dataset [23] and design a degradation model to simulate low-light images as synthetic data for training during the pre-training stage, which incorporates the novel Semantic-Guided Pixel Consistency Loss.

#### 3.2.1. Degradation Model

Compared to other image restoration tasks, the training dataset for low-light image enhancement contains a limited number of images. This scarcity leads to a high risk of overfitting and limits the models’ effectiveness in real-world applications. Furthermore, to address the difficulty of performing effective semantic segmentation caused by an excessive number of semantic categories in existing low-light datasets, we adopt the RGB500 dataset [23] with a carefully selected semantic label space, ensuring that each image contains multiple regions that are amenable to accurate segmentation, and subsequently generate synthetic low-light degraded images from it for training.

In image restoration, most existing methods [20,24] perform degradation directly on RGB images without taking the raw format into account. To achieve degradation modeling that better reflects real-world scenarios, we adopt a modified version of the approach in [25], which converts RGB images into an approximate RAW-domain representation, where the proposed degradation model is applied before converting the degraded images back to RGB. The process can be formulated as follows:

*I_RAW_* = 𝒢^−1^(CΓ^−1^(*T*^−1^(*I_RGB_*))), (2)

where *T*^−1^ and Γ^−1^ denote inverse tone mapping and gamma expansion, respectively; C represents the RGB-to-camera color correction matrix; and 𝒢^−1^ denotes inverse white balance and gain adjustment. 

This conversion process inherently increases the complexity of the added degradation distribution, making the synthesized low-light data more closely resemble real-world low-light images. Following the imaging model in [21], the formation of a digital sensor raw image *D* ∈ ℝ*^W^*^×^*^H^*^×3^ can be expressed as a linear model in the raw domain:

*D* = *K*(*I_low_* + *N_P_*) + *N_o_*, (3)

where *K* ∈ ℝ^1×1×3^ represents the overall system gain composed of analog and digital gains; *I_low_* ∈ ℝ*^W^*^×*H*×3^ is the number of photoelectrons that is proportional to the scene irradiance; *N_P_* ∈ ℝ*^W^*^×*H*×3^ denotes the photon shot noise caused by an inevitable uncertainty in the number of electrons collected due to the quantum nature of light. Given that (*I_low_* + *N_P_*) follows a Poisson distribution:

(*I_low_* + *N_P_*)~*P*(*I_low_*), (4)

where *P* denotes the Poisson distribution. *N_o_* ∈ ℝ*^W^*^×*H*×3^ accounts for other noise independent of light, which can be considered to approximately follow a Gaussian distribution.

We now consider the formation process of a low-light digital sensor raw image *I_low_*, which can be generally formulated as

*I_low_* = *L*·*I*, (5)

where *L* ∈ ℝ^1×1×3^ denotes a brightness scaling factor, and *I* ∈ ℝ*^W^*^×*H*×3^ denotes a normal-light image. Based on the above imaging model, we reformulate the degradation process to simulate low-light conditions as follows:

(6) $$D=K^{\prime }I+N_{P}^{\prime }+N_{o},$$

*K′* = *K*·*L*, (7)

and

(8) $$N_{P}^{\prime }=K\cdot N_{P},$$

where *K′* ∈ ℝ^1×1×3^ denotes the product of *K* ∈ ℝ^1×1×3^ and *L*. As *L* is intentionally sampled from the range (0.01, 0.1), the resulting *K′* is correspondingly confined to low values. This deliberate design obviates the need for camera-specific calibration of overall system gain *K*. Instead, we uniformly draw *K′* from the same interval (0.01, 0.1) to approximate the gain characteristics of various cameras, thereby enriching the training data. $N_{P}^{\prime }$ denotes Poisson noise amplified by overall system gain *K*, which is approximated using a randomly sampled noise scale to simulate variations across different camera systems. The degradation scheme of Poisson and Gaussian noise follows the same formulation as in [20]. It is worth noting that all the degradation processes are performed in the raw domain, after which the simulated raw images need to be converted back to the RGB domain.

#### 3.2.2. Semantic-Guided Pixel Consistency Loss

Due to the mismatch between the predefined label spaces of closed-set semantic segmentation models and the diverse semantic categories present in image restoration datasets, even advanced models may produce inaccurate segmentation results when applied to these datasets. Hence, we employ SAM 3 [26], an open-set segmentation model capable of performing prompt-based segmentation, to generate semantic maps for the training images. It is worth noting that the semantic segmentation maps are generated offline using SAM 3 before training and are subsequently used to compute the Semantic-Guided Pixel Consistency Loss. To maintain pixel consistency of regions with the same semantics, we propose the Semantic-Guided Pixel Consistency Loss, which can be described as

(9) $$L_{\mathrm{sg}}=\sum_{c}\frac{w_{c}}{N_{c}}{\left\|I_{\mathrm{en}}^{c}-I_{h}^{c}\right\|}_{1},$$

where *c* denotes one of the semantic labels, $I_{\mathrm{en}}^{c}\in R^{W\times H\times 3}$ denotes the pixels of the enhanced image *I_en_* ∈ ℝ*^W^*^×^*^H^*^×3^ within the corresponding semantic region in the *c^th^* semantic map. $I_{h}^{c}\in R^{W\times H\times 3}$ denotes the pixels of the ground truth image *I_h_* ∈ ℝ*^W^*^×^*^H^*^×3^ within the corresponding semantic region in the *c^th^* semantic map. *N_c_* denotes the total number of pixels assigned to semantic category *c*. *w_c_* denotes the *c^th^* weight of each semantic label, which can be described as

*w_c_* = 1 + 0.2*c*, *c* ∈ {1, 2, …, *n_c_*}, (10)

where *w_c_* are defined using a linear weighting strategy, with the coefficient 0.2 determining the interval between semantic label weights to ensure sufficient differentiation among semantic regions. *n_c_* denotes the total number of semantic labels. This design assigns different gradient contributions to semantic regions during optimization, thereby encouraging the network to learn region-dependent restoration behaviors.

The overall training objective consists of three components: the L1 reconstruction loss, the feature consistency loss, and the proposed Semantic-Guided Pixel Consistency Loss. The reconstruction loss is defined as

(11) $$L_{\mathrm{rec}}=\frac{1}{N}{\left\|I_{\mathrm{en}}-I_{h}\right\|}_{1},$$

where *N* is the total number of image elements.

During the pre-training stage, all three loss functions are employed to jointly supervise the network. The overall pre-training objective is formulated as follows:

ℒ*_pre_* = ℒ*_rec_* + ℒ*_align_* + ℒ*_sg_*. (12)

During the fine-tuning stage, we employ real low-light image datasets that predominantly contain indoor scenes with diverse objects, making it difficult to establish a unified semantic label space for reliable segmentation. Therefore, semantic segmentation maps are not utilized during fine-tuning, and the Semantic-Guided Pixel Consistency Loss is excluded. The fine-tuning objective is defined as follows:

ℒ*_fine_* = ℒ*_rec_* + ℒ*_align_*. (13)

## 4. Experimental Results and Discussion

### 4.1. Experiment Settings

#### 4.1.1. Datasets and Metrics

We evaluate our method on four LLIE benchmark datasets, including LOL(v1 [3] and v2 [27]), LIME [28], and MEF [29]. The LOL dataset has v1 and v2 versions. In contrast to LOLv1, LOLv2 is divided into real and synthetic subsets. LOLv1 contains 485 training pairs and 15 test pairs. LOLv2-real contains 689 training pairs and 100 test pairs, whereas LOLv2-synthetic contains 900 training pairs and 100 test pairs. In contrast, LIME and MEF are real-world datasets containing unpaired images. In addition to LLIE benchmark datasets, we use the dataset collected in [23], which contains 500 clean outdoor images. For paired datasets, we use peak signal-to-noise ratio (PSNR), structural similarity index measure (SSIM) [30] and learned perceptual image patch similarity (LPIPS) [31]. For unpaired datasets, we use NIQE [32], BRISQUE [33] and MANIQA [34].

#### 4.1.2. Implementation Details

The training process consists of two stages, with synthetic pre-training followed by fine-tuning on the corresponding benchmark datasets. During pre-training, we use all 500 clean outdoor images from the RGB500 dataset [23] to generate paired synthetic low-light images using the proposed degradation model. Semantic segmentation maps are generated offline from the clean images using SAM 3 to provide semantic-aware supervision. During fine-tuning, the pre-trained model is further optimized separately on the training sets on LOLv1, LOLv2-real, and LOLv2-synthetic, without semantic segmentation supervision.

Our method is implemented in PyTorch version (2.8.0). We conduct our experiments on a single NVIDIA 4090D GPU. No customized weight initialization strategy is employed, and the fine-tuning stage is initialized with the weights obtained from pre-training. In both training stages, the training images are randomly cropped into 192 × 192 patches without additional data augmentation, and the model is trained using the Adam optimizer (*β*_1_ = 0.9, *β*_2_ = 0.999). In each stage, the initial learning rate is set to 1 × 10^−4^ and then steadily decreases to 0 using a cosine annealing schedule. Both pre-training and fine-tuning are conducted for 4 × 10^5^ iterations with a batch size of 8. Unless otherwise specified, the results reported in the main experiments are obtained from a single training run. Additional experiments across six independent runs with different random seeds are presented in Appendix A.4 to evaluate training stability.

### 4.2. Quantitative Evaluation

Quantitative results on LOL datasets are shown in Table 2. Compared with other state-of-the-art methods (Zero-DCE [35], RUAS [36], MIRNet [37], SNRNet [10], PairLIE [38], LLFormer [39], Retinexformer [11] and CIDNet [40]), our method exhibits superior performance. Specifically, for distortion metrics, our method outperforms all state-of-the-art methods in terms of PSNR, achieving performance improvements of 0.3 dB, 0.74 dB and 0.87 dB in the LOLv1, LOLv2-real and LOLv2-synthetic datasets, respectively. Our method also achieves higher SSIM values than all competing methods, with improvements of 0.0139, 0.0125 and 0.0125 in the LOLv1, LOLv2-real and LOLv2-synthetic datasets, respectively. In terms of the perceptual metric LPIPS, our method achieves lower values than all competing methods, with reductions of 0.0212, 0.0096, and 0.0150 on the LOLv1, LOLv2-real, and LOLv2-synthetic datasets, respectively.

Meanwhile, we conducted comparative evaluations with competing methods on two unpaired datasets, MEF and LIME, to validate the efficacy of our method in terms of visual quality by employing three no-reference perceptual metrics, NIQE, BRISQUE and MANIQA. As shown in Table 3, we achieve the best performance across all quantitative assessments for both BRISQUE and MANIQA, while for NIQE, we also have the second-best results. These results collectively indicate that our proposed method can generate restored images while ensuring satisfactory visual quality, further demonstrating the effectiveness of our method.

For a fair comparison, we evaluate the competing methods in Table 2 using their publicly available pre-trained models on the same benchmark test sets. All quantitative metrics are calculated under a consistent evaluation protocol. Additionally, we reproduce representative semantic-guided methods whose pre-trained weights are not publicly available. These methods are evaluated using the same restoration backbone to ensure a fair comparison, and the corresponding quantitative results are presented separately in Appendix A.1.

### 4.3. Qualitative Evaluation

**Figure 4:**
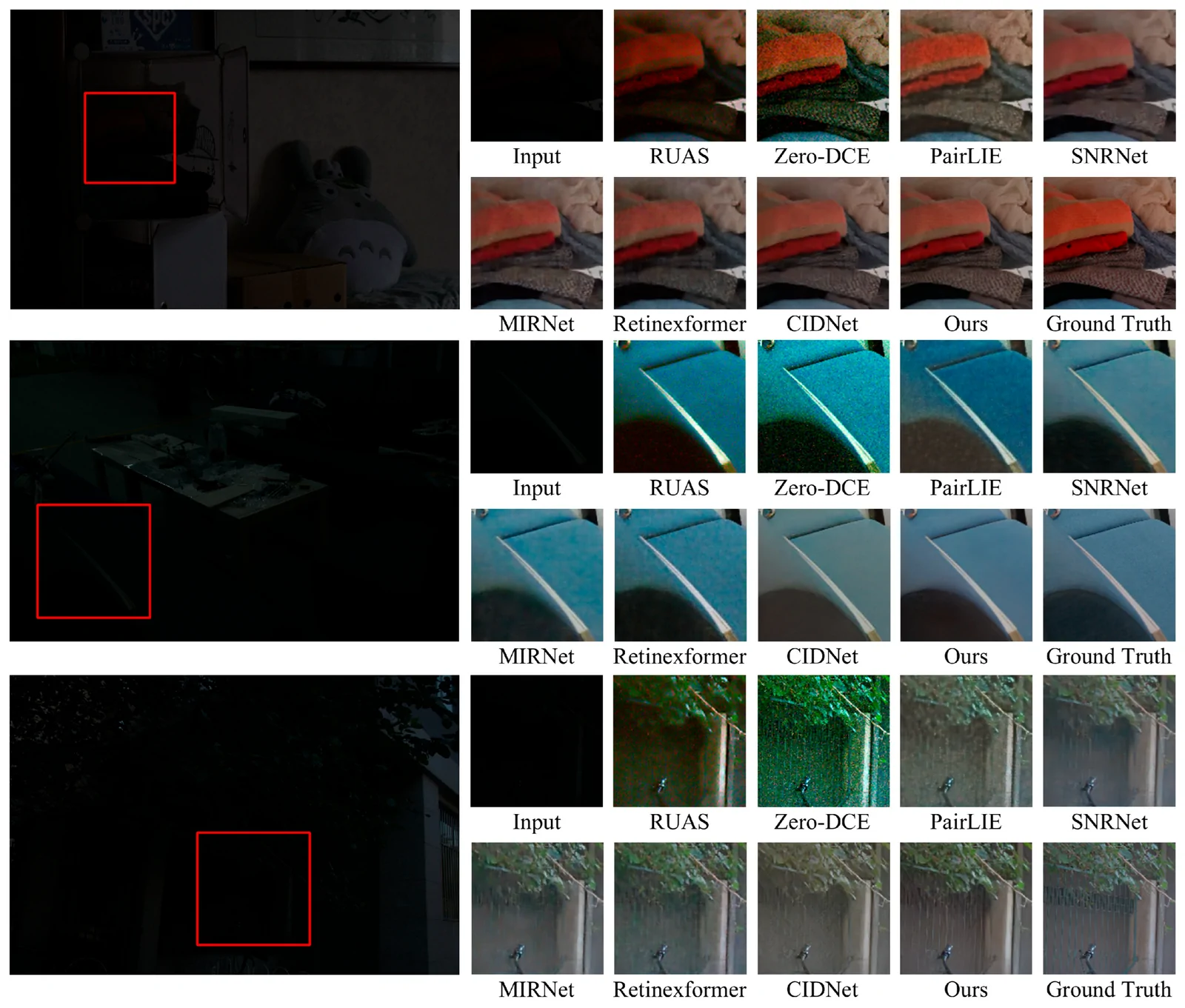
Visual results on the LOLv1 (row 1) and LOLv2-real (rows 2 and 3) test sets. Our method achieves effective noise suppression while preserving and restoring fine-grained textures.

Figure 4 presents a visual comparison between our method and state-of-the-art (SOTA) methods. Previous methods exhibit different limitations: RUAS and Zero-DCE introduce color distortions, while PairLIE, Retinexformer, and MIRNet fail to adequately suppress noise, and CIDNet introduces unnatural artifacts. In contrast, our method effectively improves visibility and contrast in low-light regions while suppressing noise and preserving color fidelity without introducing noticeable artifacts. Furthermore, our method preserves richer texture details and, despite the varying noise distributions across different semantic regions, performs region-adaptive noise suppression, effectively preventing over-smoothing in certain areas while avoiding insufficient denoising in others.

**Figure 5:**
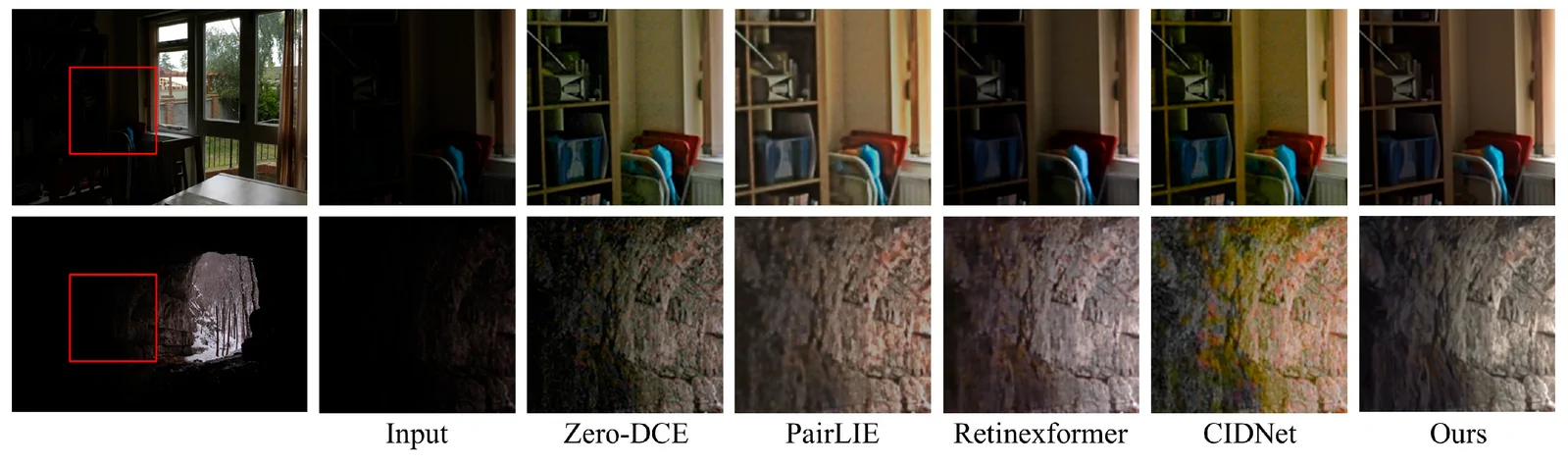
Visual results on the unpaired MEF dataset. Our method demonstrates effective noise suppression and color distortion correction.

Figure 5 presents a visual comparison on the MEF dataset. Our model effectively suppresses noise in dark regions while restoring more natural overall color tones. These results further demonstrate the effectiveness of our method on the evaluated real-world low-light images, suggesting its potential applicability to diverse low-light scenarios.

### 4.4. Ablation Study

We conduct ablation studies on the LOLv1 dataset to demonstrate the effectiveness of our method from various aspects.

#### 4.4.1. Implicit Guidance Ablation

As shown in Table 4, we conduct experiments on the LOLv1 dataset to investigate the contribution of each basic technique of the implicit semantic guidance branch. The addition of the CLIP image encoder increases the PSNR by 0.19 dB and SSIM by 0.0049 over the baseline, along with a 0.0064 reduction in LPIPS. Incorporating LM further improves the model’s performance, yielding improvements of 0.33 dB in PSNR and 0.0060 in SSIM over the baseline, along with a 0.0095 reduction in LPIPS. By jointly employing LM and FAM to enhance the semantic features extracted by the CLIP image encoder, we achieve improvements of 0.84 dB in PSNR and 0.0064 in SSIM over the baseline, along with a 0.035 reduction in LPIPS.

#### 4.4.2. Explicit Guidance Ablation

As shown in Table 4, we conduct experiments on the LOLv1 dataset to investigate the contribution of each basic technique of the explicit semantic guidance branch. By pre-training on synthetic data generated by the degradation model and fine-tuning on the LOLv1 dataset, the model achieves a 0.85 dB improvement in PSNR and a 0.0111 improvement in SSIM, along with a 0.0165 reduction in LPIPS. By jointly exploiting the implicit semantic guidance branch and the degradation model, we achieve improvements of 0.9 dB in PSNR and 0.0112 in SSIM over the baseline, along with a 0.039 reduction in LPIPS. Finally, jointly employing the implicit and explicit semantic guidance branches further improves the model’s performance, yielding improvements of 0.99 dB in PSNR and 0.0128 in SSIM over the baseline, along with a 0.0434 reduction in LPIPS.

Meanwhile, we conduct experiments on the LOLv1 dataset to investigate the contribution of the semantic label weight *w_c_* in the Semantic-Guided Pixel Consistency Loss ℒ*_sg_*. As shown in Table 4 and Table 5, *w_c_* plays a critical role in the effectiveness of ℒ*_sg_*. In the absence of *w_c_*, the results with and without ℒ*_sg_* are nearly identical, while incorporating *w_c_* leads to consistent improvements in PSNR, SSIM and LPIPS.

Finally, a detailed performance-complexity analysis, including comparisons across different model configurations and a parameter-matched baseline, is provided in Appendix A.2. Furthermore, additional ablation experiments on different benchmark datasets are provided in Appendix A.3 to investigate the effectiveness and generalizability of the proposed components.

#### 4.4.3. Hybrid Explicit–Implicit Semantic Guidance Framework

To investigate the applicability of our semantic guidance framework to other models, we conduct experiments on the LOLv1 dataset to evaluate whether other baselines can obtain consistent performance improvements under this framework. As shown in Table 6, consistent improvements are achieved over both baseline methods, NAFNet [41] and Restormer [42]. Specifically, our hybrid explicit–implicit semantic guidance framework yields a 0.31 dB gain in PSNR and a 0.0138 gain in SSIM over the baseline, along with a 0.0378 reduction in LPIPS for Restormer, and yields a 0.99 dB gain in PSNR and a 0.0128 gain in SSIM over the baseline, along with a 0.0434 reduction in LPIPS for NAFNet.

## 5. Conclusions

In this work, we investigated low-light image enhancement from a semantic perspective and showed that effective semantic guidance depends not only on the availability of semantic priors, but also on their reliability under severe degradation. To this end, we proposed a hybrid explicit–implicit semantic guidance framework that exploits semantic information in complementary forms. The implicit branch improves the reliability and compatibility of semantic features through the Light-up Module and Feature Alignment Module, while the explicit branch introduces semantic-region-aware supervision based on accurate segmentation maps and synthetic low-light data. Together, these designs enable the network to move beyond globally uniform enhancement and learn restoration behaviors that are better adapted to different semantic regions. Extensive experiments demonstrate consistent improvements in image quality, noise suppression, and structural preservation across the evaluated datasets.

Several limitations remain. First, the current semantic weighting strategy in Semantic-Guided Pixel Consistency Loss is relatively simple and does not explicitly account for differences in restoration difficulty, semantic importance, or uncertainty across regions. Second, relying exclusively on RGB500 for synthetic pre-training may introduce scene distribution bias and increase the risk of overfitting, potentially limiting generalization to unseen scenes. Third, the effectiveness of the implicit branch remains dependent on the robustness of the pre-trained semantic encoder. Under extremely severe underexposure or noise corruption, substantial information loss may compromise semantic feature extraction. This limitation is particularly pronounced when the original scene information is irreversibly lost. Representative failure cases and detailed analysis are provided in Appendix A.5.

Future work will focus on several directions. We will investigate uncertainty-aware and degradation-aware semantic weighting strategies to better exploit region-specific semantic information, develop more realistic and diverse low-light degradation models to improve synthetic-to-real generalization, and explore stronger visual foundation models as semantic priors. We also plan to extend the proposed framework to low-light video enhancement, where temporal consistency provides an additional source of semantic guidance. Finally, we will examine whether the explicit–implicit semantic guidance paradigm can generalize beyond LLIE to other ill-posed restoration tasks, with the broader goal of establishing semantic information as a general prior for content-adaptive image restoration.

## Figures and Tables

**Table 1:** Comparison of parameter counts and computational complexity between the Transformer self-attention module and the proposed Light-up Module (LM). FLOPs are calculated for an RGB input image of size 192 × 192.

Method	Params (M)	FLOPs (G)
Self-Attention	0.0060	0.2193
Light-up Module	0.0034	0.1011
Reduction	43.33%	53.90%

**Table 2:** Quantitative comparison of the LOLv1 and LOLv2 datasets. Higher PSNR and SSIM values and lower LPIPS values indicate better performance.

Method	Reference	LOLv1	LOLv2-Real	LOLv2-Synthetic
		PSNR/SSIM/LPIPS	PSNR/SSIM/LPIPS	PSNR/SSIM/LPIPS
Zero-DCE	CVPR’20	20.95/0.5338/0.3555	20.49/0.5347/0.3559	21.46/0.8505/0.1493
RUAS	CVPR’21	18.66/0.5213/0.2580	18.94/0.5093/0.2981	17.87/0.6969/0.3423
MIRNet	PAMI’23	26.55/0.8562/0.1284	28.02/0.8737/0.1604	28.69/0.9414/0.0543
SNRNet	CVPR’22	26.81/0.8520/0.1504	27.35/0.8711/0.1505	27.82/0.9416/0.0504
PairLIE	CVPR’23	23.56/0.7549/0.2389	24.07/0.8026/0.2276	21.65/0.8201/0.2238
LLFormer	AAAI’23	26.13/0.8299/0.1652	27.88/0.8718/0.1606	28.57/0.9402/0.0510
Retinexformer	ICCV’23	27.18/0.8501/0.1286	27.74/0.8566/0.1660	29.04/0.9385/0.0554
CIDNet	CVPR’25	26.67/0.8595/0.1045	28.01/0.8828/0.1161	28.18/0.9327/0.0589
Ours	-	27.48/0.8734/0.0833	28.76/0.8953/0.1065	29.91/0.9541/0.0354

The best result is in red, while the second-best result is in blue.

**Table 3:** Quantitative comparisons of MEF and LIME datasets.

Dataset	Method	NIQE	BRISQUE	MANIQA
MEF	Zero-DCE	3.282	16.67	0.4010
	PairLIE	4.193	29.80	0.3216
	Retinexformer	4.335	14.26	0.3146
	CIDNet	3.744	23.05	0.3746
	Ours	3.332	13.43	0.4116
LIME	Zero-DCE	3.771	18.52	0.4102
	PairLIE	4.526	25.39	0.3509
	Retinexformer	4.062	15.36	0.4054
	CIDNet	4.127	18.88	0.4244
	Ours	3.838	15.08	0.4268

The best result is in red, while the second-best result is in blue.

**Table 4:** Ablation study of the proposed components.

NAFNet	CLIP	LM	FAM	DM	𝓛*_sg_*	PSNR	SSIM	LPIPS
√						26.49	0.8606	0.1267
√	√					26.68	0.8655	0.1203
√	√	√				26.82	0.8666	0.1172
√	√	√	√			27.33	0.8670	0.0917
√				√		27.34	0.8717	0.1102
√	√	√	√	√		27.39	0.8718	0.0877
√	√	√	√	√	√	27.48	0.8734	0.0833

**Table 5:** Ablation study for investigating the contribution of *w_c_*.

𝓛*_sg_*	*w_c_*	PSNR	SSIM	LPIPS
√		27.38	0.8710	0.0912
√	√	27.48	0.8734	0.0833

**Table 6:** Ablation study for investigating the contribution of hybrid explicit–implicit semantic guidance.

Method	PSNR	SSIM	LPIPS
NAFNet	26.49	0.8606	0.1267
NAFNet + (Ours)	27.48	0.8734	0.0833
Restormer	26.91	0.8601	0.1204
Restormer + (Ours)	27.22	0.8739	0.0826

**Figure A1:**
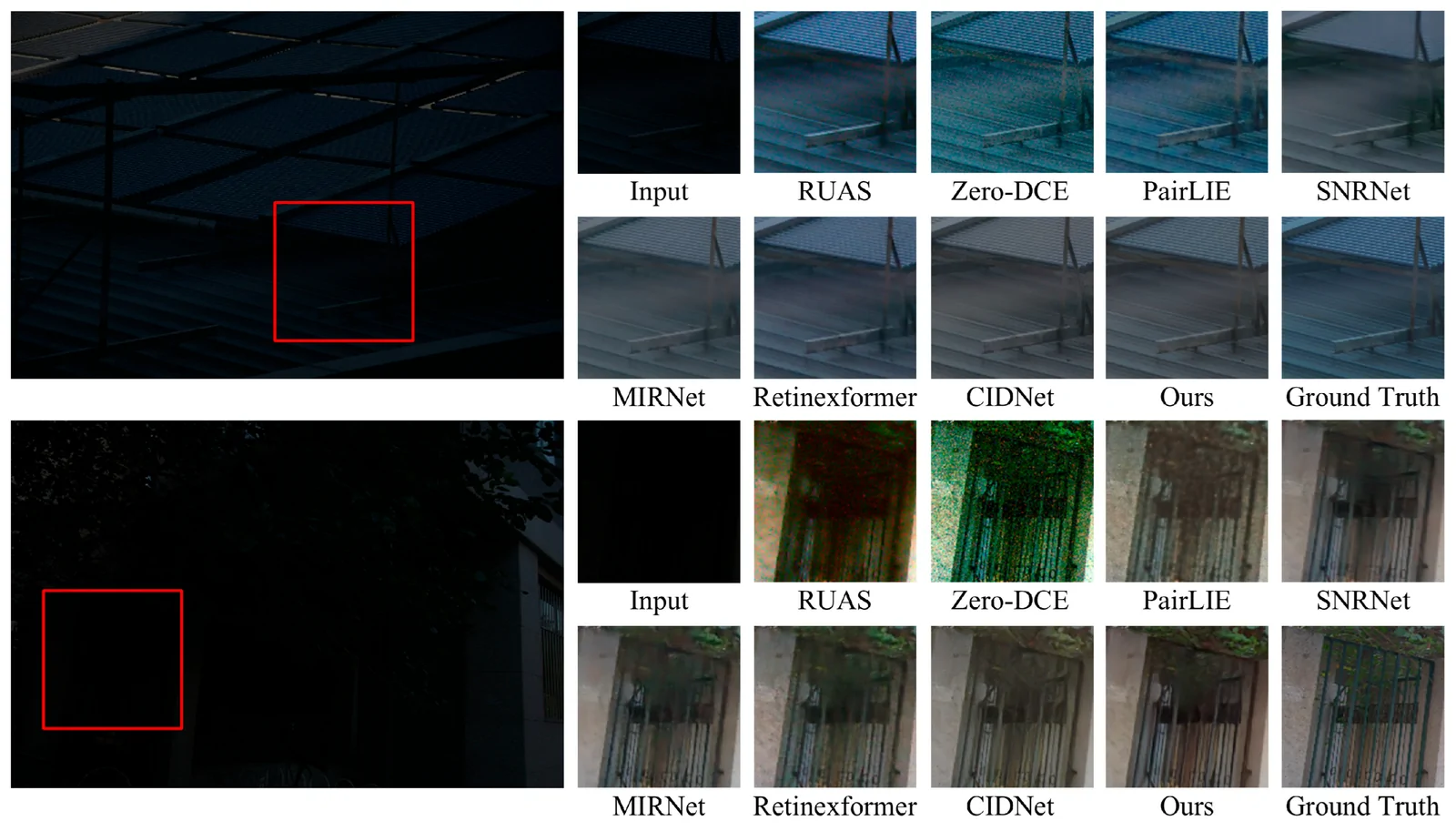
Visual comparisons of representative failure cases of the proposed method.

## Data Availability

The datasets analyzed in this study, including LOLv1, LOLv2, MEF, RGB500 and LIME, are publicly available from their respective sources, as cited in the paper.

## Abbreviations

The following abbreviations are used in this manuscript:

LLIE	Low-light Image Enhancement
LM	Light-up Module
FAM	Feature Alignment Module
DM	Degradation Model
OOD	Out-of-distribution
CLIP	Contrastive Language-Image Pre-Training
SAM	Segment Anything Model
PSNR	Peak Signal-to-Noise Ratio
SSIM	Structural Similarity Index Measure
LPIPS	Learned Perceptual Image Patch Similarity
NIQE	Natural Image Quality Evaluator
BRISQUE	Blind/Referenceless Image Spatial Quality Evaluator
MANIQA	Multi-dimension Attention Network for Image Quality Assessment

## Appendix A

### Appendix A.1

To further evaluate the proposed hybrid explicit-implicit semantic guidance framework against existing semantic-guided approaches, we conduct additional comparisons with SKF [6] and SeD [7], the latter adapted from image super-resolution to low-light image enhancement. As shown in Table A1, our method consistently outperforms both approaches across PSNR, SSIM and LPIPS on LOLv1 dataset.

**Table A1:** Quantitative comparison of different semantic guidance methods using NAFNet as the backbone on the LOLv1 dataset. Higher PSNR and SSIM values and lower LPIPS values indicate better performance.

Methods	PSNR	SSIM	LPIPS
NAFNet	26.49	0.8606	0.1267
NAFNet-SKF	26.68	0.8655	0.1111
NAFNet-SeD	26.55	0.8531	0.1041
NAFNet-Ours	27.48	0.8734	0.0833

### Appendix A.2

Table A2 presents the parameter counts, FLOPs, and restoration performance of different model configurations. The semantic-aware embedding module (SEM) adopted from [6] is employed to integrate semantic features into the restoration network. Incorporating LM and FAM progressively improves restoration quality, while semantic-guided supervision introduces no additional inference cost. Our framework outperforms the enlarged baseline with a comparable parameter count, achieving a 0.37 dB improvement in PSNR, a 0.0085 increase in SSIM, and a 0.0320 reduction in LPIPS, while requiring 30.3% fewer FLOPs. These results demonstrate the effectiveness of the proposed framework in balancing restoration performance and computational complexity.

**Table A2:** Performance-complexity comparison of different model configurations on the LOLv1 dataset. FLOPs are calculated at an input resolution of 192 × 192.

Model	Params (M)	FLOPs (G)	PSNR	SSIM	LPIPS
Baseline	64.64	33.05	26.49	0.8606	0.1267
+CLIP&SEM	96.95	39.35	26.68	0.8655	0.1203
+LM	96.95	39.45	26.82	0.8666	0.1172
+FAM	124.95	43.38	27.33	0.8670	0.0917
Baseline *	122	62.27	27.11	0.8649	0.1153
Ours	124.95	43.38	27.48	0.8734	0.0833

Baseline * denotes the enlarged baseline.

### Appendix A.3

To further investigate the effectiveness and generalizability of the proposed components, we conduct additional ablation experiments on LOLv2-real, following the same experimental configurations as those presented in Section 4.4. The quantitative results are presented in Table A3. These additional experiments provide further evidence regarding the effectiveness of the proposed hybrid explicit-implicit semantic guidance framework across different low-light datasets.

**Table A3:** Ablation study of the proposed components on different datasets.

NAFNet	Imp	Exp	LOLv1			LOLv2-Real		
			PSNR	SSIM	LPIPS	PSNR	SSIM	LPIPS
√			26.49	0.8606	0.1267	27.61	0.8732	0.1489
√	√		27.33	0.8670	0.0917	28.32	0.8876	0.1169
√		√	27.33	0.8731	0.1086	28.06	0.8783	0.1449
√	√	√	27.48	0.8734	0.0833	28.76	0.8953	0.1065

Imp denotes the Implicit semantic guidance branch. Exp denotes the Explicit semantic guidance branch.

### Appendix A.4

To further evaluate the stability of the proposed framework, we conduct six independent training runs using different random seeds. All training configurations remain unchanged except for random seeds.

As shown in Table A4, the proposed method achieves an average PSNR of 27.44 dB, an SSIM of 0.8738, and an LPIPS of 0.0830, with corresponding standard deviations of 0.0361 dB, 0.0004, and 0.0005, respectively. The relatively small standard deviations indicate that the proposed framework maintains consistent restoration performance across different random seeds, demonstrating its training stability.

**Table A4:** Quantitative results of the proposed method across six independent training runs with different random seeds on the LOLv1 dataset.

Metrics	Seed 1	Seed 2	Seed 3	Seed 4	Seed 5	Seed 6	Mean	Std.
PSNR	27.38	27.44	27.43	27.47	27.46	27.48	27.44	0.0361
SSIM	0.8735	0.8740	0.8734	0.8745	0.8740	0.8734	0.8738	0.0004
LPIPS	0.0829	0.0836	0.0823	0.0833	0.0829	0.0833	0.0830	0.0005

### Appendix A.5

To further investigate the limitations of the proposed framework, we present representative failure cases in Figure A1. The first row illustrates a challenging case involving semantic categories not represented during semantic-aware pre-training, where the limited coverage of semantic priors may restrict the effectiveness of semantic guidance. Although the proposed method effectively mitigates low-light degradation and demonstrates superior performance in texture restoration and noise suppression, it fails to accurately recover the original color information.

The second row shows a case of severe low-light degradation, where substantial information loss compromises semantic feature extraction and limits the recovery of image details. Although the corresponding semantic categories were included during semantic-aware pre-training, the severe degradation causes significant loss of semantic information in the input image. Consequently, the proposed method fails to recover meaningful scene information even after enhancement.

These examples highlight the limitations of the proposed framework in handling unseen semantic categories and extremely degraded regions.
